# A rhesus macaque intragastric challenge model for evaluating the safety, immunogenicity, and efficacy of live-attenuated *Shigella dysenteriae* 1 vaccine candidates

**DOI:** 10.3389/fmicb.2024.1454338

**Published:** 2024-09-06

**Authors:** Nattaya Ruamsap, Rawiwan Imerbsin, Patchariya Khanijou, Siriphan Gonwong, Wilawan Oransathit, Shoshana Barnoy, Malabi M. Venkatesan, Sidhartha Chaudhury, Dilara Islam

**Affiliations:** ^1^Department of Bacterial and Parasitic Diseases, Armed Forces Research Institute of Medical Sciences, Bangkok, Thailand; ^2^Department of Veterinary Medicine, Armed Forces Research Institute of Medical Sciences, Bangkok, Thailand; ^3^Department of Diarrheal Disease Research, Bacterial Disease Branch, Walter Reed Army Institute of Research, Silver Spring, MD, United States

**Keywords:** *Shigella dysenteriae* 1, WRSd1, WRSd2, WRSd3, WRSd4, WRSd5, live attenuated vaccines, rhesus monkey

## Abstract

Shigellosis remains a significant global health challenge, particularly in Asia and Africa, where it is a major cause of morbidity and mortality among children. Despite the urgent need, the development of a licensed *Shigella* vaccine has been hindered, partly due to the lack of suitable animal models for preclinical evaluation. In this study, we used an intragastric adult rhesus macaque challenge model to evaluate the safety, immunogenicity, and efficacy of five live-attenuated *Shigella dysenteriae* 1 vaccine candidates, all derived from the 1617 parent strain. The vaccine strains included WRSd1, a previously tested candidate with deletions in virG(icsA), stxAB, and fnr, and four other strains—WRSd2, WRSd3, WRSd4, and WRSd5—each containing deletions in virG and stxAB, but retaining fnr. Additionally, WRSd3 and WRSd5 had further deletions in the *Shigella* enterotoxin gene senA and its paralog senB, with WRSd5 having an extra deletion in msbB2. Rhesus monkeys were immunized three times at two-day intervals with a target dose of 2 × 10^10^ CFU of the vaccine strains. Thirty days after the final immunization, all monkeys were challenged with a target dose of 2 × 10^9^ CFU of the *S. dysenteriae* 1 1617 wild-type strain. Safety, immunogenicity, and efficacy were assessed through physical monitoring and the evaluation of immunologic and inflammatory markers following immunization and challenge. Initial doses of WRSd1, WRSd3, and WRSd5 led to mild adverse effects, such as vomiting and loose stools, but all five vaccine strains were well tolerated in subsequent doses. All strains elicited significant IgA and IgG antibody responses, as well as the production of antibody-secreting cells. Notably, none of the vaccinated animals exhibited shigellosis symptoms such as vomiting or loose/watery stool post-challenge, in stark contrast to the control group, where 39% and 61% of monkeys exhibited these symptoms, respectively. The aggregate clinical score used to evaluate *Shigella* attack rates post-challenge revealed a 72% attack rate in control animals, compared to only 13% in vaccinated animals, indicating a relative risk reduction of 81%. This study highlights the potential of this NHP model in evaluating the safety, immunogenicity, and efficacy of live-attenuated *Shigella* vaccine candidates, offering a valuable tool for preclinical assessment before advancing to Phase 1 or more advanced clinical trials.

## 1 Introduction

Shigellosis, or “bacillary dysentery,” is one of the leading causes of bacterial diarrheal infections worldwide, with more than 120 million cases annually, particularly in Asia and Africa. The burden of shigellosis among children in the developing world is still a major concern. *Shigella* is a highly virulent pathogen, orally transmitted through contaminated food and water, and has a low multiplicity of infection. *Shigella* spp. comprises four major serogroups, *S. dysenteriae, S. flexneri, S. boydii, S. sonnei*, and *S. flexneri*, that contain multiple types and subtypes. *Shigella* infections are characterized by acute inflammatory colitis elicited by bacterial invasion of the intestinal epithelium (Levine et al., 1973; Sansonetti, 2008).

*S. dysenteriae* serotype 1 (Sd1) is usually seen during epidemics and in places like refugee camps with limited resources where subjects are suddenly overcrowded (Lampel et al., 2018). Earlier, it has been shown that the seasonal increase in dysentery was largely due to multidrug-resistant Sd1 (Sansonetti, 2008), and the majority (76%) of the Sd1 cases result in hemolytic uremic syndrome (Levine et al., 1973). In infants and young children, hyponatremia toxic megacolon is usually associated with Sd1 infections, and intestinal obstruction can be seen in severe cases (Ashkenazi and Cohen, 2013; Mani et al., 2016). Mucosal invasion of *Shigella* organisms can lead to rectal prolapse and proctitis (Barry et al., 2013). Sd1 was previously seen during epidemics when the disease symptoms were exacerbated by the presence of the Shiga toxin in Sd1 strains, and 5–15% of the Sd1 infections in developing country/refugee populations are fatal due to the presence of the Shiga toxin (WHO, 1996) and are still a potential problem if an outbreak occurs.

Vaccination is a common strategy to control, eliminate, eradicate, or contain infectious disease and one of the most cost-effective public health interventions. However, after several decades of investigation, there are still no broadly effective vaccines against Shigellosis. Progress in vaccine development for *Shigella* has been challenging for several reasons, such as the absence of firm correlates of protective immunity, a limited understanding of the mechanism governing protection, or lack of commercial interest. Nevertheless, currently, two major vaccine strategies for *Shigella* are being explored: live attenuated oral and subunit parenteral vaccines (Ashkenazi and Cohen, 2013; Barry et al., 2013; Mani et al., 2016). Parenteral vaccine candidates have been largely based on *Shigella* LPS O-antigen conjugates and candidates still under development and testing include the S4V-EPA vaccine by Limmatech (Clarkson et al., 2021), the SF2a-TT15 vaccine by the Pasteur Institute (Cohen et al., 2021), the Invaplex vaccine by WRAIR (Turbyfill et al., 2022), and the *Shigella* outer membrane vesicle vaccine by GSK (Micoli et al., 2022). These new vaccine candidates have demonstrated efficacy in mouse models of infection and, in some cases, in Phase 1 studies, have reached safety and immunogenicity endpoints.

Live attenuated vaccines (LAV) have a number of advantages as a vaccine platform, including following the path of natural infections, stimulating durable immunity, typically not requiring adjuvants, and being relatively cheap to manufacture and easy to deliver. The design of *Shigella* live-attenuated strains that retain the invasiveness of mammalian cells but are unable to spread from host cell to host cell has proven to be a successful strategy for the development of live vaccine candidates such as SC602 (*S. flexneri*) from the Pasteur Institute (Coster et al., 1999), WRSS1 (*S. sonnei*) from WRAIR (Kotloff et al., 2002), the CVD-1233 SP (*S. sonnei*) from the University of Maryland (Pilla et al., 2021), and the ShigETEC vaccine (*S. flexneri*) by Eveliqure (Girardi et al., 2022). Clinical trials with these vaccines have demonstrated that a single oral dose, ranging from 10^3^ to 10^4^ colony-forming units (CFU), provided safety and immunogenicity and, in some cases, efficacy against controlled human infection challenge (Coster et al., 1999; Kotloff et al., 2002; Katz et al., 2004; Orr et al., 2005; McKenzie et al., 2008).

The WRSd1 vaccine candidate is an *S. dysenteriae* vaccine based on the deletion of the *virG*(*icsA*) gene encoded on the virulence plasmid (Venkatesan et al., 2002). WRSd1 was constructed from Sd1 strain 1617 that was originally isolated during the 1968–1969 epidemic of Shiga dysentery in Guatemala (Mendizabal-Morris et al., 1971). Besides the loss of the *virG(icsA)* gene, WRSd1 contained a 20 kb chromosomal deletion that included *stxA* and *stxB* genes encoding the Shiga toxin, phage genes, and the fumarate nitrate reductase (*fnr*) gene (fumarate nitrate reductase) (McDonough and Butterton, 1999; Venkatesan et al., 2002). The loss of the *fnr* gene was an unexpected event that occurred during the construction of WRSd1 and probably contributed negatively to the shedding characteristics and limited immunogenicity observed during a phase 1 clinical trial (Venkatesan et al., 2002).

Animal models are important tools for assessing vaccine candidates for preclinical studies. Two models used for evaluating *Shigella* vaccine candidates are the murine pulmonary challenge model (Mallett et al., 1993) and the guinea pig keratoconjunctivitis model (Hartman et al., 1991). While both models are useful for early-stage evaluation of vaccine candidates, neither mimics the diarrheal disease characteristic of shigellosis. A more recent guinea pig rectocolitis model (Shim et al., 2007) does produce bloody mucoidal stools but still faces the limitations of small animal models in capturing human immunogenicity due to host immunological differences between guinea pigs and humans. Shigellosis in Rhesus macaque (*Macaca mulatta*) closely mimics the human disease and immune responses. Although the oral infective dose of *Shigella* in man is only 100–1,000 bacteria. The corresponding dose administered to monkeys is 10^9^-10^11^ CFU. Clinical features and gross microscopic lesions of the monkey colon are indistinguishable from human *Shigella* infections (Levine et al., 1973). Earlier, two *Shigella* challenge models in monkeys were established at AFRIMS by using the Sd1 strain 1617 (Islam et al., 2014, 2016) and *S. flexneri* 2a (Sf2a) strain 2457T (Islam et al., 2023). In the Sd1 challenge model, it was revealed that a previous infection with Sd1 strain 1617 protected monkeys against a subsequent challenge with the same organism (Islam et al., 2014). The Sd1 challenge model was utilized to assess the therapeutic efficacy of an oral Toll-like receptor-4 antagonist, dendrimer glucosamine, in preventing cytokine storms and severe gut wall damage (Islam et al., 2016). We recently published a preclinical evaluation of Travelan^®^ as immunoprophylactic in the developed Sf2a challenge model (Islam et al., 2023). Travelan^®^ showed compelling evidence of 75% efficacy in preventing shigellosis (Islam et al., 2023). Apart from rhesus macaque, an *Aotus* monkey model for shigellosis was also developed, which recapitulates important disease and immune characteristics of the human disease (Gregory et al., 2014), including gut colonization, diarrhea, and serum antibody responses following oral administration of both the wild-type Sf2a 2457T strain and the *Shigella* vaccine strain SC602.

In this pilot study with small groups of rhesus macaques, several *virG(icsA)* mutant Sd1 strains, all lacking the ability to spread intercellularly, including WRSd1, were compared for safety, immunogenicity, and efficacy. The strains tested included a *fnr*+ version of WRSd1 (WRSd2 and WRSd4) and a couple of closely related strains that additionally lacked the *Shigella* enterotoxin gene *senA*, its paralog *senB* (WRSd3 and WRSd5), and a lipid A-modifying enzyme *msbB2* (WRSd5). The goal was to validate the earlier study with Sd1 strain 1617 (Islam et al., 2014) and to see whether the losses of *senA, senB*, and/or *msbB2* against a strong attenuating feature could be distinguished in this model.

## 2 Materials and methods

### 2.1 Animals and ethics statement

Adult rhesus macaques (*Macaca mulatta*), either male or female, of Indian origin, were born and housed at AFRIMS. Prior to inclusion in the study, rhesus macaques were screened, and those animals meeting the inclusion criteria (seronegative for tuberculosis, simian immunodeficiency virus, simian retrovirus, simian T-lymphotropic virus type I, negative stool cultures for enteric pathogens, IgG titers of ≤ 200, in good health by physical examination, and complete blood count (CBC) and blood chemistry (BC) values within the normal range) were randomized to the various treatment groups. The study was performed under a protocol approved by AFRIMS's Institutional Animal Care and Use Committee (IACUC). All of the monkeys were observed by animal technicians certified by the American Association of Laboratory Animal Science (AALAC). Research was conducted in compliance with the U.S. Food and Drug Administration Good Laboratory Practice Regulations, 21 CFR Part 58.

### 2.2 Anesthesia/euthanasia methods

The NHP was anesthetized by an intramuscular injection of 5–20 mg of ketamine. For the euthanasia method, the NHP was chemically restrained with an intramuscular injection of ketamine hydrochloride (20 mg/kg). Once sedated, an injectable commercial euthanasia agent, Fatal-Plus (Vortech Pharmaceuticals, Ltd., MI, USA), was administered intravenously to induce euthanasia. The euthanizing dose of Fatal-Plus was 86.7 mg sodium pentobarbital/kg (0.22 ml/kg). The first half of the dose was administered rapidly, with the second half administered more slowly. The animals were observed for a few minutes until breathing ceased. The complete absence of heartbeats was confirmed using a stethoscope before the needle was removed. If the heartbeat persisted, an additional ¼ dose of the euthanasia or anesthetic agent was administered.

### 2.3 *S. dysenteriae* 1 strains

The Sd1 strains used in this study were constructed at WRAIR with loss of the *virG(icsA)* and *stxAB* genes as the primary attenuating features. During the construction of WRSd1, a 20 kb deletion occurred that removed the *fnr* gene on the chromosome that was located close to the *stxAB* genes. To produce the next series of strains, the *virG(icsA)* gene was deleted from the parent strain 1617 using the lambda red recombination method to produce WRSd4, and the *stxAB* genes were deleted using the suicide vector technique to produce WRSd2. WRSd2 and WRSd4 are *fnr* positive. WRSd2 was further engineered to delete the plasmid-encoded enterotoxin gene *senA* and its paralog *senB* to generate WRSd3 as well as the *msbB2* gene to yield WRSd5 using lambda red recombination.

Master cell banks (MCB) of Sd1 strains were prepared at AFRIMS. All Sd1 strains, including 1617, were characterized by performing the following tests: (i) a slide agglutination test with commercially available *S. dysenteriae* 1 specific antisera (*Shigella dysenteriae*, polyvalent A, type 1 antisera, Denka Seiken Co., Ltd.), (ii) HEp-2 cell invasion assay, and (iii) PCR assays to confirm the presence or the absence of specific genes. DNA templates for PCR were prepared using a heat lysis method, as described previously (Englen and Kelley, 2000). Additionally, strain 1617 was subjected to a plaque assay in LLC-MK2 cells to evaluate its spreading ability (Oaks et al., 1985; Islam et al., 2014).

### 2.4 Study design

Supplementary Figure S1 illustrates a diagrammatic representation of the study design and sample collection days. A total of 48 rhesus monkeys (RM) were selected based on inclusion and exclusion criteria and randomized into eight groups (six monkeys per group). The study was conducted in three phases. In the 1st phase, WRSd1 was evaluated with a control (unimmunized but challenged) group. In the 2nd phase, WRSd2 and WRSd4 were evaluated with a control group, and in the 3rd phase, WRSd3 and WRSd5 were evaluated with a control group. RM was intragastrically immunized on study days 0, 3, and 6 with a dose in the range of 2 × 10^10^ to 3 × 10^10^ CFU of the respective Sd1 strains. Control monkeys were received PBS on each immunization day. On study day 37 (30 days after the last immunization), all animals were intragastrically challenged with the dose ranging from 2 × 10^9^ to 3 x 10^9^ CFU of Sd1 strain 1617. RM were fasted overnight before inoculation and were anesthetized with ketamine hydrochloride before nasogastric placement. Each inoculation was preceded by nasogastric administration of 20 ml of saturated sodium bicarbonate to neutralize gastric acidity. The vaccine dose of 10^10^ was assigned based on our preliminary study (unpublished data) in evaluating multiple doses of Sd1 WRSd1 on the established rhesus monkey model of shigellosis. It was shown that multiple doses of Sd1 WRSd1 administered at 2x10^10^ CFU on days 0, 3, and 6 were safe without developing a severe clinical illness, and the WRSd1-immunized monkeys showed 80% protective efficacy against shigellosis after a challenge on day 37 with 2x10^10^ CFU of Sd1 1617. The challenge dose of Sd1 1617 was followed (Islam et al., 2014), in which 2 x 10^9^ CFU was identified as an optimal challenge dose.

Blood and fecal samples were collected pre-immunization as a baseline, post-immunization, and post-challenge. Following the challenge, any animals that developed severe diarrhea and/or dysentery will be treated with enrofloxacin dose 5 mg/kg body weight (Baytril^®^) (Bayer Korea Ltd., Korea) *via* intramuscular injection once daily for 7 days consecutively. All monkeys that did not receive early antibiotic treatment due to adverse events were administered antibiotics 15 days after the challenge and were humanely euthanized on day 66 (30 days after the challenge). During the study, RM was monitored daily for any symptoms that suggested an adverse reaction, including diarrhea, fever, vomiting, reduced activity, recumbency, allergic reactions (e.g., swelling, itchiness, and rash), or anaphylactic reactions (e.g., collapse, anemia, hypotension, tachypnea, dyspnea, hypothermia, tremors, seizure, and urinary incontinence).

### 2.5 Stool samples

Stool specimens were collected twice daily (morning and afternoon) to assess the shedding of the Sd1 vaccine and challenge strains. All stools were evaluated for the presence of blood or mucus and for fecal consistency. The shedding of Sd1 was evaluated using both standard culture procedures and a real-time PCR assay. Fecal secretory IgA (s-IgA) and fecal cytokines (Islam et al., 2014) were also measured in fecal extract samples. Fecal samples were extracted with an extraction solution as described earlier (Islam et al., 2006, 2014).

For the real-time PCR assay, DNA templates were prepared from stool specimens using the QiaAmp Stool Purification Kit (Qiagen, Germantown, MD, USA). These purified DNA templates were then used in a real-time PCR reaction to detect the presence of *Shigella* species by targeting the *ipaH* marker, as outlined in previous studies (Hartman et al., 1990; Vu et al., 2004; Islam et al., 2014, 2016). After each immunization, a conventional PCR assay using multiple primers was also conducted to characterize the shedding of the Sd1 strains.

### 2.6 Blood samples

CBC was assessed using the SYSMEX XT-2000i automated hematology analyzer (Sysmex America, Inc., IL, USA). BC was analyzed using the COBAS^®^ C111 automated analyzer (Roche Diagnostics, IN, USA). The procedures were performed according to the manufacturer. Blood samples for CBC and BC were collected on the day (−35±10).

Blood samples collected on the day (−3) pre-immunization, days 3, 6, 10, 13, and 20 post-immunizations, day 34 (pre-challenge), and days 40, 44, 47, 51, 55, and 65 post-challenge were used to separate serum for evaluating antibody responses by ELISA. Blood samples collected on days (-3) pre-immunization, day 10 post-immunizations, day 34 (pre-challenge), and day 44 post-challenge were used to separate the fresh peripheral blood mononuclear cells (PBMCs) for determining the antibody-secreting cells (ASCs) responses by using enzyme-linked immunospot (ELISPOT) assay.

### 2.7 *S. dysenteriae* 1 antigens

Immune responses were evaluated using the following antigens: Sd1 LPS and Sd1 Invaplex-24 (INV) (Oaks et al., 1996; McKenzie et al., 2008). Sd1 LPS was prepared from WRSd1 (Commonwealth Biotechnologies, Inc.). Sd1 INV is a water extract of the Sd1 strain, which contains invasion plasmid antigens (Ipa) (predominantly IpaB and IpaC) and LPS (McKenzie et al., 2008) and was a gift from Dr. Edwin V. Oaks at WRAIR.

### 2.8 Humoral immune responses

As described previously, serum immune responses were measured using standard ELISA to detect IgA, IgG, and IgM antibody titers against Sd1 LPS and INV antigens (Islam et al., 2014). Antibody titers were determined by 4-parameter analysis using Soft Max-Pro software (Molecular Devices, LLC). Seroconversion was defined as a >4-fold rise in antibody titers compared to the baseline levels.

### 2.9 Fecal secretory IgA (s-IgA) responses

*Shigella* antigen-specific s-IgA responses in fecal extracts were determined using the ELISA procedure (Islam et al., 2014). A ≥ 4-fold rise in antibody titers compared to baseline was considered a positive response.

### 2.10 Antibody-secreting cell assay

Mucosal responses were also measured as Sd1 antigen-specific ASCs by ELISPOT assay and analyzed using a CTL ImmunoSpot^®^ Analyzer (Cellular Technology Limited). The ASCs were expressed per 10^6^ PBMCs (Van de Verg et al., 1990; Islam et al., 2014). A positive ASC response was defined as a count greater than the mean plus three standard deviations (> MN+3SD) of the baseline observed on day 10 (after three immunizations) and on day 44 (after the challenge). For baseline ASC counts that were equal to 0, a positive ASC response was defined as ≥10 ASCs per 10^6^ PBMCs on days 10 and 44.

### 2.11 Measurement of cytokines in fecal extract samples

Cytokine levels of interleukin (IL)-1β, IL-6, and IL-8 were measured in fecal extract samples using a bead-based immunoassay system (Luminex Corporation), which can quantitate multiple cytokines simultaneously at the protein level (Oliver et al., 1998; Vignali, 2000).

### 2.12 Determination of white blood cell inflammatory score (WIS)

To access and compare inflammatory changes induced by the Sd1 strains and protection against inflammation after the challenge, each RM received a WIS score (formulated by the Department of Veterinary Medicine, AFRIMS) post-immunization and post-challenge. The WIS was calculated as follows:

WIS = (highest band neutrophils/ml)/(1,000 cells/μl) + No. of days toxic change present. For each time period, the WIS from all individual animals was averaged to give a group average.

### 2.13 Clinical disease assessment

A clinical disease assessment index was developed by the Department of Veterinary Medicine at AFRIMS to quantify the relative degree of morbidity in rhesus macaques (RM) following the administration of Sd1 live strains. The clinical scores assigned, detailed in Supplementary Table S1, were based on the “Guidelines on the Recognition of Pain” (Levine, 2010). All animals were closely observed and scored at least twice daily using a clinical observation report. To measure body temperature, remote sensing transponder thermometers (IPTT-200, Bio Medic Data System, Inc.) were placed subcutaneously in the RMs.

The clinical parameters assessed included activity, appetite, fecal mucus and/or blood, and skin turgor. The total clinical score determined the health status of the monkeys:

- **Score** **<5**: Normal health.
- **Score 5-9**: Animals required close monitoring and might be provided with electrolytes in their drinking water.
- **Score 10-14**: Animals were provided with electrolytes or intravenous fluids, and analgesics were considered.
- **Score** **≥15**: The monkeys were evaluated for euthanasia. However, based on veterinary guidance, some RMs were euthanized with lower clinical scores.

Dysentery was defined as the presence of at least one watery or loose stool containing blood and mucus. Disease attack was defined as reaching a total clinical score of ≥5 within seven days after challenge with the 1,617 strain. Disease was indicated either by physical observation or through clinical laboratory results in both immunized and control monkeys.

### 2.14 Data analysis

Data analyses were performed using IBM SPSS Statistics, version 26. ELISA data (titers) were presented as geometric mean titers with standard errors of the means (GMN ± SEM). The cytokine concentrations of the samples were calculated using xPONENT^®^ software (Luminex Corporation). Standard curves for the various cytokines were constructed by a 4-parameter regression formula and plotted as a linear curve (log-log) according to the manufacturer's instructions. Unless mentioned otherwise, a pairwise comparison of groups was conducted using the Independent-Samples Kruskal-Wallis Test. Vaccine efficacy was measured as the relative risk reduction of attaining a total *Shigella* clinical score of ≥ 5 in the vaccinated groups relative to the control group. Moreover, *p* < 0.05 were considered statistically significant.

## 3 Results

### 3.1 Genotypic characterization and virulence properties of the Sd1 strains

WRSd1 was constructed from parent strain 1617 and has been previously described and evaluated in a phase 1 trial (McKenzie et al., 2008). WRSd4 was constructed from the same parent strain as WRSd1 to ensure that the *fnr* gene was retained with the loss of *virG(icsA)* and then further engineered into WRSd2 by the deletion of the *stxAB* genes (Table 1). WRSd3 and WRSd5 lacked *senA* and *senB* genes encoded on the virulence plasmid, and WRSd5 also missed the *msbB2* gene (Table 1). All the Sd1 strains were strongly positive on Congo Red (CR) agar plates, and the stability of the strains was checked by subculturing them. WRSd1, WRSd2, WRSd3, WRSd4, and WRSd5 strains were positive on CR plates and in invasion assays and negative in plaque assays and in the sereny test in guinea pig eyes. Unlike WRSd1, the remaining Sd1 strains used in this study, WRSd2, WRSd3, WRSd4, and WRSd5, were positive for the *fnr* gene (Table 1).

**Table 1:** Live, attenuated oral Sd1 vaccine strains (WRSd1, WRSd2, WRSd3, WRSd4, and WRSd5) contain deletions in different gene segments identified by PCR assay with different primers.

**Genes**	**Primers**	**Size (bp)**	**1,617**	**WRSd1**	**WRSd2**	**WRSd3**	**WRSd4**	**WRSd5**
*ipaH7.8*	IpaH7.8	509	+	+	+	+	+	+
*virA*	VirA	899	+	-	+	+	+	+
*stxAB*	Stx100	1,354	+	-	-	-	-	-
*virG(icsA)*	VirG(icsA)	1,000	+	-	-	-	-	-
*shET2-1*	SenA	723	+	+	+	-	+	-
*shET2-2*	SenB	750	+	-	+	-	+	-
*msbB2*	MsbB2	749	+	+	+	+	+	-
*fnr*	Fnr	849	+	-	+	+	+	+
*stxB*	StxB	202	+	-	-	-	+	-

“+”, positive PCR with the specific primers; “-”, negative PCR with the specific primers.

The number of animals/groups is six monkeys for each of the Sd1 vaccine strains and 18 monkeys for the Sd1 1617 challenge strain (six monkeys/phase; a total of six phases were run).

### 3.2 Safety of the Sd1 vaccine strains in the rhesus macaque model

The primary endpoint for the safety of each Sd1 LAV was diarrhea (loose or watery stool) and vomiting. Except for the monkeys in the WRSd2 and WRSd4 groups, vomiting post-immunization was seen in 2–3 monkeys in the WRSd1, WRSd3, and WRSd5 groups. Monkeys belonging to WRSd1, WRSd3, and WRSd5 groups also had non-dysenteric loose stools in 1–2 animals only after the 1st dose, except for one monkey in the WRSd5 group that had loose stools also after the 2nd dose (Table 2). The animals in the WRSd2 group demonstrated neither vomiting nor loose stool after immunization. Immediate adverse anaphylactic reactions were not seen in any of the immunized monkeys. Monkeys with loose stools had higher clinical scores and required metoclopramide, acetar, and/or electrolyte in drinking water. As expected, the control RMs receiving PBS during the immunization phase were healthy.

**Table 2:** Percentage of monkeys in each group that experienced adverse effects within 24 h after each immunization.

**Group**	**Vomiting (%)**			**Loose/watery stool (%)**			**Clinical score of** ≥**5 (%)**		
	**Dose 1**	**Dose 2**	**Dose 3**	**Dose 1**	**Dose 2**	**Dose 3**	**Dose 1**	**Dose 2**	**Dose 3**
WRSd1	3/6 (50)	0	0	2/6 (33)	0	0	1/6 (17)	0	0
WRSd2	0	0	0	0	0	0	0	0	0
WRSd3	2/6 (33)	0	0	1/6 (17)	0	0	1/6 (17)	0	0
WRSd4	0	0	0	0	0	0	0	0	0
WRSd5	2/6 (33)	0	0	2/6 (33)	1/6 (17)	0	0	0	0
All vaccinated	7/30 (23)	0	0	5/30 (17)	1/30 (3)	0	2/30 (7)	0	0
Control	0	0	0	0	0	0	0	0	0

The number of animals/groups is six monkeys for each of the Sd1 vaccine strains and 18 monkeys for the Sd1 1617 challenge strain (six monkeys/phase; a total of three phases were run).

### 3.3 Efficacy in vaccinated monkeys after challenge with the strain 1617

The major clinical effects following exposure to the Sd1 1617 strain included loose, watery stool with blood and mucus (dysentery). The most common constitutional symptom noted was significant appetite loss (AL, defined as consumption of < 50% of normal food intake, Table 3). Loss of appetite is a sensitive indicator of digestive distress with enteric pathogens. Sixteen of 18 (89%) control monkeys had appetite loss, and so did 2–4 immunized monkeys in each group, with the WRSd5 group of monkeys showing the highest percentage (67%) of animals with appetite loss post-challenge (Table 3). No vomiting was noted in any group of immunized monkeys after the challenge, while vomiting was noted in 7 of 18 (39%) control monkeys (Table 3). Dysentery was noted in only one animal each in the WRSd1, WRSd2, and WRSd3 groups post-challenge with clinical scores of 5–9. No dysentery was noted in the WRSd5 group after the challenge. None of the immunized monkeys after the challenge required early antibiotic treatment prior to the study's scheduled time. On the other hand, 11 of 18 control monkeys (61%) had dysentery after the challenge and a clinical score of ≥ 5. Eight of the control monkeys had a score of 10–14 and required metoclopramide and Buprenex to relieve pain in addition to early antibiotic treatment. The number of monkeys with adverse reactions (vomiting and dysentery) and clinical scores of ≥ 5 within seven days after the challenge are indicated in Table 3. The control attack rate after the 1617 challenge based on a clinical score of ≥ 5 was 72%. In general, monkeys require oral supplements, such as fluid IV injections, acetar, and electrolytes in drinking water. The protective efficacy estimated for each group of animals after the challenge indicates that the WRSd5 immunized group had the best protection (100%), followed closely by the WRSd1, WRSd2, and WRSd3 immunized groups, which provided comparable levels of protection (Table 3).

**Table 3:** Vaccine efficacy and percentage of monkeys in each group that experienced shigellosis symptoms within seven days of challenge.

**Group**	**Loss of appetite (%)**	**Vomiting (%)**	**Loose/watery stool (%)**	**Clinical score of ≥5 (%)**	**Clinical score of ≥10 (%)**	**Vaccine efficacy (%)**
WRSd1	2/6 (33)	0	1/6 (17)	1/6 (17)	0	76
WRSd2	2/6 (33)	0	1/6 (17)	1/6 (17)	0	76
WRSd3	3/6 (50)	0	1/6 (17)	1/6 (17)	0	76
WRSd4	2/6 (33)	0	0	1/6 (17)	0	76
WRSd5	4/6 (66)	0	0	0	0	100
All vaccinated	13/30 (43)	0	3/30 (10)	4/30 (13)	0	82
Control	16/18 (89)	7/18 (38)	11/18 (61)	13/18 (72)	8/18 (44)	N/A

Vaccine efficacy percentages were determined based on a clinical score of only ≥ 5.

The number of animals/groups is six monkeys for each of the Sd1 vaccine strains and 18 monkeys for the Sd1 1617 challenge strain (six monkeys/phase; a total of three phases were run).

White blood cell changes typically seen in cases with shigellosis include leukocytosis, neutrophilia with left shift (presence of immature neutrophils such as band cells in circulation), and toxic change in neutrophils often seen as cytoplasmic Döhle bodies (retained aggregates of rough endoplasmic reticulum) (Fried et al., 1982; Halpern et al., 1992). These changes indicate an acute inflammatory response. Table 4 shows the white blood cell inflammatory score (WIS) of monkeys in each group, both after immunization and after challenge. The percentage increase of immature neutrophils, known as the “left shift,” may happen when an acute infection stimulates increased neutrophil production. A left shift is usually accompanied by a toxic change in neutrophils, and toxic changes are measured by the presence of Döhle bodies. Döhle bodies were not observed in any of the monkey neutrophils post-immunization, and as a result, the WIS score was < 1 in all immunized and control groups. However, after the challenge monkeys in the WRSd1 and WSd3 groups had WIS scores of > 1 compared to the other immunized groups, indicating inflammatory changes. Döhle bodies were observed in 13 of 18 (69%) control monkeys after the challenge with 1617. However, the average WIS score in the control group was lower compared to the WIS score in the WRSd1 and WRSd3 groups. The WIS score post-challenge was significantly different in controls compared to the WRSd1 group (*p* = 0.031) and the WRSd3 group (*p* = 0.033).

**Table 4:** WIS score of monkeys at post-immunization and post-challenge in each group.

**Monkey groups**	**No. of monkeys with DB**	**Average WIS**	
	**PC**	**PI**	**PC**
WRSd1	6	0.10	2.62
WRSd2	2	0.07	0.89
WRSd3	3	0.05	2.48
WRSd4	4	0.05	0.88
WRSd5	2	0.06	0.43
Control	13	0.11	1.70

PI, Post-immunization; PC, Post-challenge; DB, Döhle bodies (presence of DB indicates toxic changes). DB was absent in all monkeys after immunization. The WIS was calculated for each monkey. The number of animals/groups is six monkeys for each of the Sd1 vaccine strains and 18 monkeys for the Sd1 1617 challenge strain (six monkeys/phase; a total of three phases were run).

### 3.4 Shedding of the Sd1 vaccine and challenge strains

**Figure 1:**
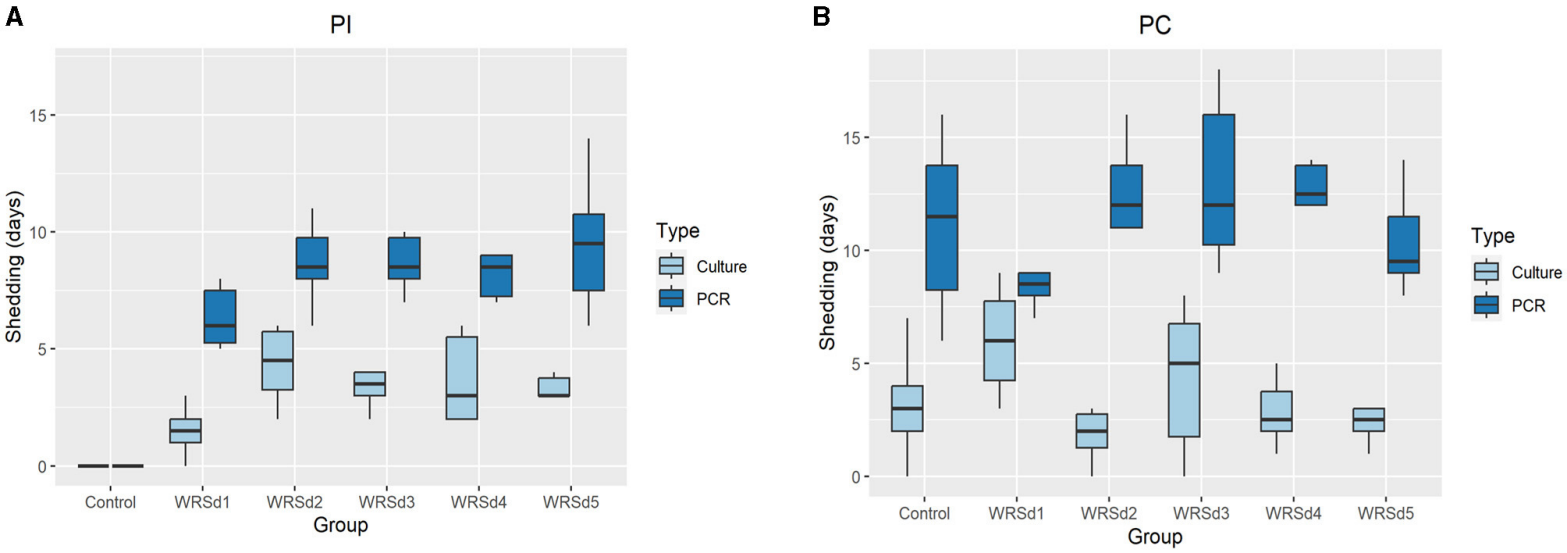
Days of fecal shedding of Sd1 were measured by stool culture (light blue) and PCR (dark blue) for control and vaccinated groups. **(A)** Post-immunization, **(B)** post-challenge. Box and whisker plots represent the mean from six vaccinated monkeys and 18 control monkeys. The number of animals/groups is six monkeys for each of the Sd1 vaccine strains and 18 monkeys for the Sd1 1617 challenge strain (six monkeys/phase; a total of three phases were run).

We measured fecal shedding as the number of days following immunization and challenged that Sd1 was detectable in the stool-by-stool culture and PCR (Figure 1). WRSd1 was the least able to colonize the monkeys compared to other immunized groups. For WRSd1, five of six monkeys showed shedding by stool culture following immunization, while for the other vaccine groups, all six monkeys showed shedding. Regarding average days shedding, the WRSd1 group showed significantly less shedding (*p* < 0.01) compared to the other groups, with an average number of shedding days of 1.5 days, compared to 4.3, 3.3, 3.6, and 3.7 days. This difference was also significant when measuring shedding by PCR (*p* < 0.05). No other significant differences in shedding were observed between the vaccine groups following immunization. Following the challenge, nearly 96% of all animals (46 of 48) showed shedding in both the vaccine and control groups, and there was no significant difference in shedding between the groups by both stool culture and PCR. It is important to note that early antibiotic treatment of 44% (8 of 18) control monkeys may have affected the shedding of the Sd1 strain in this group.

### 3.5 Systemic and mucosal immune responses at post-immunization and post-challenge

**Figure 2:**
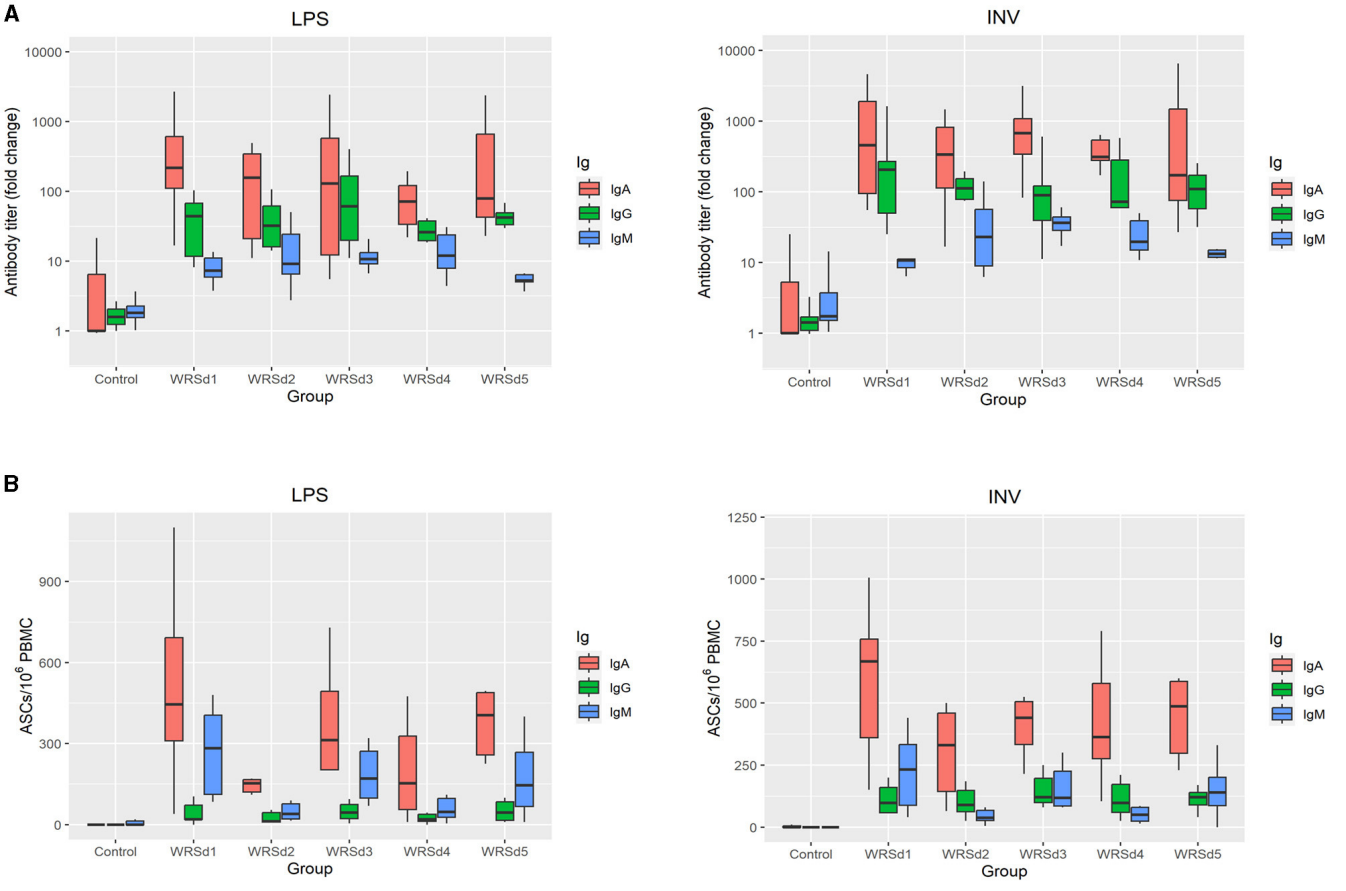
Systemic and mucosal responses to Sd1 LPS and INV antigens in monkeys after three doses of vaccination for the control group and the four vaccinated groups. **(A)** Peak fold-rise IgA, IgG, and IgM antibody titers compared to baseline and **(B)** peak antigen-specific ASC/10^6^ PBMC count. Box and whisker plots represent the mean from 6 vaccinated monkeys and 18 control monkeys. The number of animals/groups is six monkeys for each of the Sd1 vaccine strains and 18 monkeys for the Sd1 1617 challenge strain (six monkeys/phase; a total of three phases were run).

All the monkeys in each of the immunized groups seroconverted with IgA, IgG, and IgM antibody responses against both Sd1 LPS and Sd1 INV antigens (Figure 2A). In general, serum IgA antibody responses were higher than IgG and IgM responses, and responses to Sd1 INV were generally higher than responses to Sd1 LPS. We found that the peak fold rise of the IgA antibody titer against Sd1 INV was significantly higher than the IgM titer for all vaccine strains. For antibodies specific to the Sd1 LPS, the IgA antibody titer level was significantly higher than the IgM antibody titer for three of the five strains (WRSd1, WRSd4, and WRSd5). Comparing the peak fold increase of IgG and IgM antibody titers, IgG titer was significantly higher than IgM titer for some cases: WRSd1 for the Sd1 INV and WRSd5 for both Sd1 LPS and INV. Among the three antibody types, the mean peak fold rise of the IgA antibody titer had the highest level. Overall, these findings indicate that each Sd1 vaccine candidate can induce a robust IgA antibody response. Regarding the kinetics of the antibody responses following immunization, the most common day with peak response was days 10, 13, and 6 for IgA, IgG, and IgM responses across all vaccine groups (Supplementary Figure S3).

Comparing the post-immune and post-challenge antibody responses, there was a significant difference in the IgM antibody titers against both LPS and INV in all the immunized groups (*p* = 0.031) (Supplementary Figures S1–S4). Less than 50% of the monkeys in each group, except for the WRSd2 group, seroconverted after challenge with a serum IgM response against both antigens. In the WRSd2 group, 67% seroconverted with an IgM response to the Sd1 INV antigen. In terms of the kinetics of antibody responses following the challenge (Supplementary Figure S3), we found that the challenge, in general, did boost antibody responses across the vaccine groups and that the most common day on which peak post-challenge antibody responses were observed was day 51, day 55, and day 47 for IgA, IgG, and IgM responses, respectively. The post-challenge antibody responses in the vaccinated groups were similar to the levels attained by the control monkeys. Pairwise comparisons of all groups showed that none of the serum response differences between immunized and control animals post-challenge were significant, except in the WRSd1 and WRSd5 groups, where the IgM antibody peak fold-rise after the challenge was significantly lower than that seen in the control group of monkeys (*p* = 0.015).

Mucosal responses to the Sd1 antigens were measured by performing the ELISPOT assay for measuring ASCs (Figure 2B). In general, IgA ASC counts against both antigens were higher than IgG ASCs. In WRSd1 and WRSd2 immunized monkeys, post-immunization and post-challenge ASCs against both antigens were not significantly different from each other or from the ASCs seen in control monkeys post-challenge. In contrast, in the WRSd3 and WRSd5 groups, LPS-specific IgA, IgG, and IgM ASCs were significantly lower post-challenge than post-immunization, and the post-challenge responses were also lower than seen in the control group (*p* = 0.015). Significantly different from IgA ASCs against LPS and IgM ASCs against both antigens (*p* = 0.031). In the WRSd1 group, INV-specific IgA and IgM responses post-challenge were comparable to the control group of animals; however, the INV-specific IgG ASC responses were significantly higher than in the control group (*p* = 0.024).

### 3.6 Fecal sIgA antibody and cytokine responses

**Figure 3:**
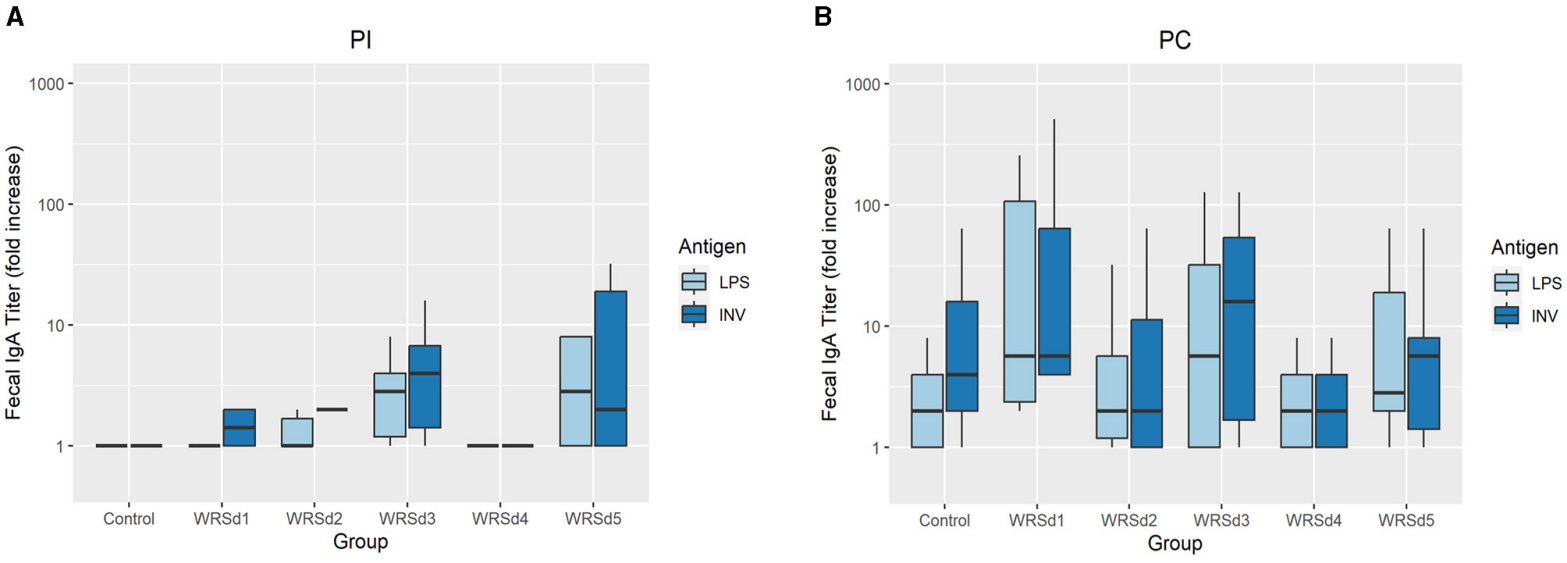
The peak fold increase from baseline in fecal IgA titers against Sd1 LPS (light blue) and INV (dark blue) for the control group and the vaccinated groups **(A)** post-immunization timepoints and **(B)** post-challenge timepoints. Box and whisker plots represent the mean from six vaccinated monkeys and 18 control monkeys. The number of animals/groups is six monkeys for each of the Sd1 vaccine strains and 18 monkeys for the Sd1 1617 challenge strain (six monkeys/phase; a total of three phases were run).

Post-immunization, fecal sIgA responses were nominal in all monkeys (data not shown). Post-challenge, fecal sIgA responses were evaluated for baseline titers on the day of challenge and 7 to 10 days post-challenge (Figure 3). The control group had the lowest LPS-specific significant rise of fecal sIgA, while the WRSd3 group had the highest fecal sIgA rise against both antigens. The WRSd5 group had the lowest significant rise of fecal sIgA response against INV, while the rest of the groups had the same level of response to both antigens. The differences in the LPS and INV-specific increase of sIgA antibody titers post-challenge among the immunized animal groups were insignificant.

**Figure 4:**
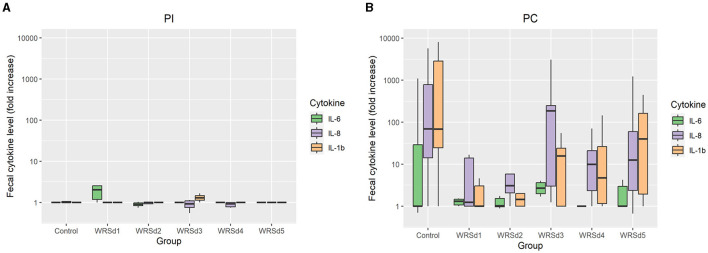
The fold change in fecal cytokine concentrations from baseline for the control and vaccinated groups. **(A)** Post-immunization time points **(B)** post-challenge time points. Green, purple, and orange represent IL-6, IL-8, and IL-1β, respectively. Box and whisker plots represent the mean from six vaccinated monkeys and 18 control monkeys. The number of animals/groups is six monkeys for each of the Sd1 vaccine strains and 18 monkeys for the Sd1 1617 challenge strain (six monkeys/phase; a total of three phases were run).

Pro-inflammatory cytokines IL-1β, IL-6, and IL-8 were measured in fecal extracts of each monkey post-immunization and post-challenge (Figure 4). The post-immunization fecal cytokine response was low in all vaccine groups, indicating that the immunization with the vaccine strain was well tolerated. For post-challenge, the IL-8 response was the highest, while the IL-6 response was the lowest in all the monkey groups. The SE was extremely high for all three cytokine levels, as the range is from 0 to >10,000 in all groups except in the WRSd5 group, where the range was from 0 to 1,200. In the control group post-challenge, the levels of all three cytokines were the highest compared to all four immunized groups. Among the vaccine groups, WRSd3 and WRSd5 groups showed the highest cytokine levels, particularly for IL-8 and IL-1β.

## 4 Discussion

In this study, we present the application of an NHP shigellosis challenge model for evaluating the safety and efficacy of *Shigella* LAVs for Sd1. The NHP model has advantages over other animal models both because it can reproduce human-like shigellosis symptoms such as gut colonization, diarrhea, and bloody mucoidal stool, as well as because the NHP immune system is a good surrogate for human immunity and reactogenicity. We tested a series of five Sd1 LAVs (WRSd1, WRSd2, WRSd3, WRSd4, and WRSd5) and found that the vaccines were generally well-tolerated, immunogenic, and protective against oral challenge with wild-type Sd1 strain 1617. We found that the LAVs successfully colonized the gut and observed shedding in the stool using both culture and PCR. We also found low levels of IgA in the stool, which was elevated following the challenge, indicating a potential recall response upon challenge. Following the oral challenge, 13 of 18 (72%) of the control animals had moderate symptoms, such as diarrhea, and 8 of 18 (44%) had severe symptoms, such as dysentery, that required early antibiotic intervention. By contrast, only 13% of vaccinated animals showed moderate symptoms, and none required early antibiotic intervention.

In earlier studies, the levels of pro-inflammatory cytokines such as IL-1β, IL-6, and IL-8 were shown to be related to *Shigella*-induced inflammation (de Silva et al., 1993; Azim et al., 1995; Raqib et al., 1995; Singer and Sansonetti, 2004). It has also been shown that significantly higher levels of pro-inflammatory cytokines peaked at the onset of severe shigellosis (Raqib et al., 1995). In a previous study, we found that monkeys that were challenged with virulent Sd1 strain 1617 were found to have mainly IL-1β, IL-8, and low levels of IL-6 secreted in the stools (Islam et al., 2014). Similarly, in this study, we found elevated levels of IL-6, IL-1B, and IL-8 following challenge in both control animals and vaccinated animals. However, in the vaccinated animals, this *Shigella*-induced inflammation did not appear to be linked with severe *Shigella* symptoms. Overall, the presence of gut colonization, elevated IgA antibody responses, and inflammatory cytokines in the stool of vaccinated challenged animals suggests that while sterile immunity was not achieved, vaccine-induced immune responses may have played some role in reducing symptom severity.

The main drawback of LAVs is typically their reactogenicity; to address safety and reactogenicity, the LAV doses and number of doses must be optimized. In this NHP study, three consecutive doses were administered 2 days apart, as the colonization of vaccine strains was not more than one or two days. To increase the colonization days, the vaccine was administered 2 days apart, with an expectation of stronger and higher immunogenicity. Vaccine doses were one log higher than the challenge dose. We showed previously that the higher the challenge dose, the better the protective efficacy and the higher the reactogenicity (Islam et al., 2014). Overall, we found limited reactogenicity, with some animals showing adverse effects of vomiting and loose stool following the first dose, but all five vaccine strains were well-tolerated in the second and third doses. Likewise, analysis of the stool showed that while gut colonization was achieved in all vaccine groups, only very low levels of inflammatory cytokines were observed in the stool post-immunization, suggesting a low inflammatory response against the vaccine strain.

McKenzie et al. (2008) tested the WRSd1 vaccine candidate in a Phase I clinical trial, inoculating 40 volunteers (8 people per study group) with a single dose of 10^3^ to 10^7^ CFU. The vaccine was safe, as none of the vaccines developed fever or shigellosis. In addition, IgA-ASC response to Sd1 LPS was presented in almost two-thirds of the vaccines. In contrast, serum IgA responses to Sd1 LPS and INV were displayed in one-third of the vaccines, but serum IgM responses to Sd1 LPS were not detected in any vaccines (McKenzie et al., 2008). McKenzie et al. also observed poor shedding of WRSd1 during the phase 1 study, indicating a lack of robust gastrointestinal colonization that they suggested as a potential reason for the low immunogenicity. The poor shedding of WRSd1 was attributed to the unexpected loss of the *fnr* gene, which is required for colonization of *E. coli* K-12 strains in an animal model (Venkatesan et al., 2002), and formed the basis for the WRSd2 to WRSd5 strains. In this study, we found robust shedding and immunogenicity for the WRSd1 strain, with all of the WRSd1-immunized monkeys eliciting IgA, IgG, and IgM responses to both Sd1 LPS and INV. We found that WRSd2 to WRSd5 had higher shedding by both culture and PCR, supporting the theory that *fnr* contributes to robust colonization. The difference in WRSd1 immunogenicity in our study and the Phase I clinical trial could be because our study used a higher dose with repeated immunization compared to McKenzie et al. (10^10^ CFU vs. the highest dose at 10^7^ CFU; a three-dose regimen vs. a single dose). It is possible that a higher dose with repeated immunization of WRSd1 could induce stronger humoral IgA, IgG, and IgM responses in humans.

A LAV *S. flexneri* 2a (Sf2a) SC602 vaccine strain is an interesting comparison as it was tested in both NHP models and in human clinical studies. SC602 was shown to have high protective efficacy in both rhesus macaques and *Aotus nancymaae* models. Rhesus monkeys with three vaccinations (days 0, 10, and 20) of 8 x 10^10^ CFU of strain SC602 had 75% protection against challenge with 1 x 10^11^ CFU of virulent Sf2a 2457T (Gregory et al., 2014). This finding was consistent with the *Aotus* monkey model, showing 80% protection after three vaccinations (days 0, 14, and 42) of 10^10^ and 10^11^ CFU of the SC602 vaccine strain (Gregory et al., 2014). In clinical trials of North American volunteers, a dose of 10^4^ CFU of the SC602 vaccine was well tolerated. It did not cause serious adverse events, whereas 50% of vaccines that received a higher dose of 10^6^ CFU experienced high reactogenicity, including adverse effects such as diarrhea or fever (Coster et al., 1999; Katz et al., 2004). In addition, the 10^4^ CFU vaccine dose of SC602 was found to be immunogenic, as shown by strong ASC responses and protection against dysentery following challenge with 10^3^ CFU of virulent Sf2a 2457T (Coster et al., 1999; Katz et al., 2004). In contrast, in clinical trials in Bangladesh, the same high-dose regimen (10^6^ CFU) was not found to be associated with reactogenicity in adults and children, and this dose failed to induce high immunogenicity (Coster et al., 1999; Katz et al., 2004; Rahman et al., 2011).

The findings from SC602 and other clinical trials of *Shigella* LAVs suggest two observations for *Shigella* vaccines. First, reactogenicity and immunogenicity may be linked, and finding a dose and/or regimen that strikes a balance between the two may be challenging. Second, the relationship between dose, reactogenicity, and immunogenicity may be linked to whether an individual is naïve or has repeated exposure due to living in an endemic area (Katz et al., 2004; Rahman et al., 2011). In the case of WRSd1 and the vaccine strains tested in this study, we observed differences in dose, reactogenicity, and immunogenicity between NHP and human studies. In NHPs, the vaccine showed modest reactogenicity and strong immunogenicity and protection at a relatively high dose, compared to the human study (McKenzie et al., 2008), where the vaccine showed low reactogenicity and low immunogenicity at a relatively low dose. Taken together, these findings highlight the challenges of using animal models for preclinical testing of *Shigella* vaccines, as the relationship between dose, reactogenicity, and immunogenicity is likely to be highly host-organism specific. Ultimately, the role of NHP models may be focused on demonstrating that inducing protective gut immunity with a candidate vaccine is possible, but dose-escalation studies in Phase 1 clinical trials are needed to determine if the right balance of reactogenicity and immunogenicity can be achieved in different types of subject populations (children vs. adults, endemic vs. non-endemic, etc.).

For the *Shigella* vaccine, the main hurdle has been the strain specificity of the various antigens under evaluation, and the significant number of candidates currently being evaluated reflects the lack of success in advancing a broad-spectrum, efficacious vaccine. Recent impact studies indicate that ETEC and *Shigella* vaccines could significantly benefit global public health, either combined together or with another enteric vaccine, would be an extremely valuable tool for saving lives and promoting the health of infants and children in the developing world, as well as potentially providing protection to travelers and military personnel visiting endemic areas (Walker, 2015). However, before a *Shigella* vaccine can become feasible, more research is required to produce either broad-spectrum or polyvalent vaccine formulations of different serotypes that can provide long-lasting immunity across *Shigella* serotypes. Further studies are required, specifically those focused on including additional strains or antigens, deleting other genes, and using different concentrations of the various vaccine candidates to obtain a broad range of vaccinations. Despite their limitations in capturing certain aspects of dose and reactogenicity, NHP *Shigella* challenge models will likely play a critical role in future studies on modeling immunogenicity and efficacy in broad-spectrum or multi-component *Shigella* vaccines.

## Data Availability

The raw data supporting the conclusions of this article will be made available by the authors, without undue reservation.

## Author disclaimer

Material has been reviewed by the Walter Reed Army Institute of Research. There is no objection to its presentation and/or publication. The opinions or assertions contained herein are the private views of the author. They are not to be construed as official or as reflecting true views of the Department of the Army or the Department of Defense. The research was conducted under an IACUC-approved animal use protocol in an AAALAC International-accredited facility with a Public Health Services Animal Welfare Assurance and in compliance with the Animal Welfare Act and other federal statutes and regulations relating to laboratory animals.

## Supplementary material

Supplementary Table S1 Assigned clinical scores.

Supplementary Figure S1 A diagrammatic representation of the study design and sample collection days.

Supplementary Figure S2 The peak fold increase in serum antibody titers against the Sd1 LPS and INV antigens over baseline for the control and five vaccinated groups. Box and whisker plots represent the mean from 6 vaccinated monkeys and 18 control monkeys. Monkeys were immunized with the Sd1 vaccine strains on days 0, 3, and 6. On day 37, all animals were challenged with the Sd1 1617 wild-type strain. No significance was observed between post-immunization and post-challenge using the Mann-Whitney test.

Supplementary Figure S3 The serum IgA, IgG, and IgM antibody titers kinetics against the Sd1 LPS and INV antigens were used for the control group and five vaccinated groups. Each symbol in each graph represents the geomean from 6 vaccinated monkeys and 18 control monkeys. Monkeys were immunized with the Sd1 vaccine strains on days 0, 3, and 6. On day 37, all animals were challenged with the Sd1 1617 wild-type strain.

Supplementary Figure S4 The fold increase in ASC responses against the Sd1 LPS and INV antigens over baseline for the control and five vaccinated groups. Box and whisker plots represent the mean from 6 vaccinated monkeys and 18 control monkeys. Monkeys were immunized with the Sd1 vaccine strains on days 0, 3, and 6. On day 37, all animals were challenged with the Sd1 1617 wild-type strain. No significance was observed between post-immunization and post-challenge using the Mann–Whitney test.

Supplementary Figure S5 Peak fold increase in IgA, IgG, and IgM antibody titers t post-challenge against Sd1 LPS and INV antigens for the five vaccinated groups. Monkeys were immunized with the Sd1 vaccine strains on days 0, 3, and 6. On day 37, all animals were challenged with the Sd1 1617 wild-type strain. The box and whisker plots represent the mean from six vaccinated monkeys. ^*^*p* < 0.05, ^**^*p* < 0.01, using the Kruskal–Wallis test followed using Dunn's *post hoc* test.
