# Supplementary material for: A rhesus macaque intragastric challenge model for evaluating the safety, immunogenicity, and efficacy of live-attenuated Shigella dysenteriae 1 vaccine candidates
Source: Front Microbiol. 2024 Sep 6;15:1454338. doi: 10.3389/fmicb.2024.1454338 (PMC11413625; doi:10.3389/fmicb.2024.1454338)
Supplement: Supplementary Table S1 — Assigned clinical scores. [file Table_1.pdf]

Supplementary Material

Table S1. Assigned clinical scores.

Criterion	Observation	Score
Activity	Active and responsive	0
	Reduced/subdued	1
	Immobile	2
	Recumbent	3
Mentation	Alert	0
	Inattentive	1
	Non-responsive	2
Breathing	Normal rate (8-15)	0
	Mild dyspnea	1
	Moderate dyspnea	2
	Gasping	3
Tremors	None present	0
	Slight shaking (as if cold)	1
	Noticeable tremors	2
	Convulsions/severe tremor	3
Vomiting	None present	0
	Retching or dry heaves	1
	Vomiting or vomitus present	2
Skin turgor	Normal (<2 sec)	0
	Return 2-4 sec	1
	Return 5-9 sec	2
	Return >10 sec	3
Feces consistency	Normal	0
	Soft but formed stool	1
	Loose stool	2
	Watery	3
Mucus/Blood	Absent	0
	Mucus present	1
	Blood present	2
	Both mucus and blood present	3
Appetite $= \frac{\text{\# biscuits eaten} \times 100}{\text{\# biscuits given}}$	≥90%	0
	≥70% to 89%	1
	≥50% to 69%	2
	<50%	3
