# Supplementary figures and images for: A rhesus macaque intragastric challenge model for evaluating the safety, immunogenicity, and efficacy of live-attenuated Shigella dysenteriae 1 vaccine candidates
Source: Front Microbiol. 2024 Sep 6;15:1454338. doi: 10.3389/fmicb.2024.1454338 (PMC11413625; doi:10.3389/fmicb.2024.1454338)

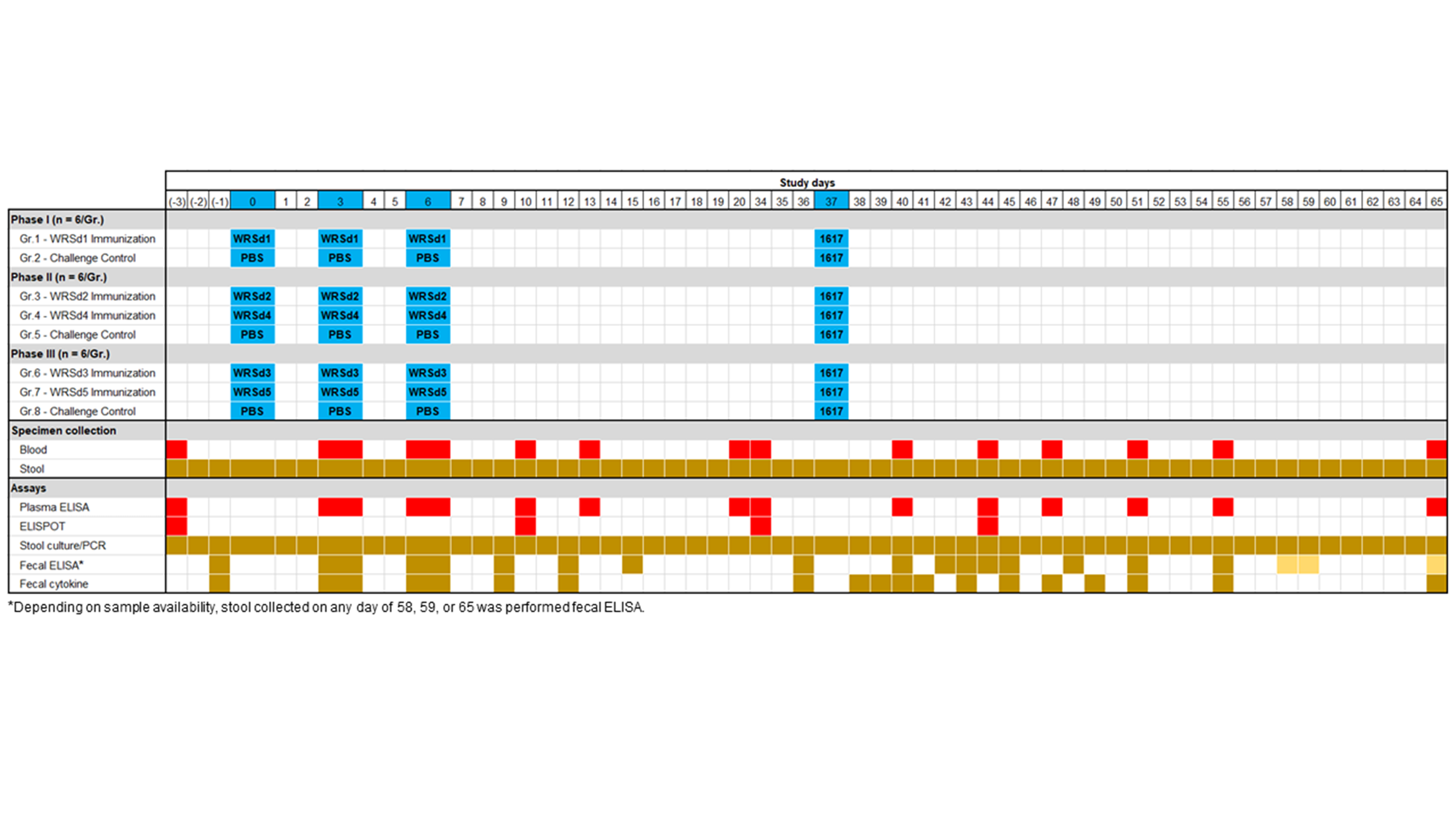

Supplement: Supplementary Figure S1 — A diagrammatic representation of the study design and sample collection days. [file Image_1.TIF]

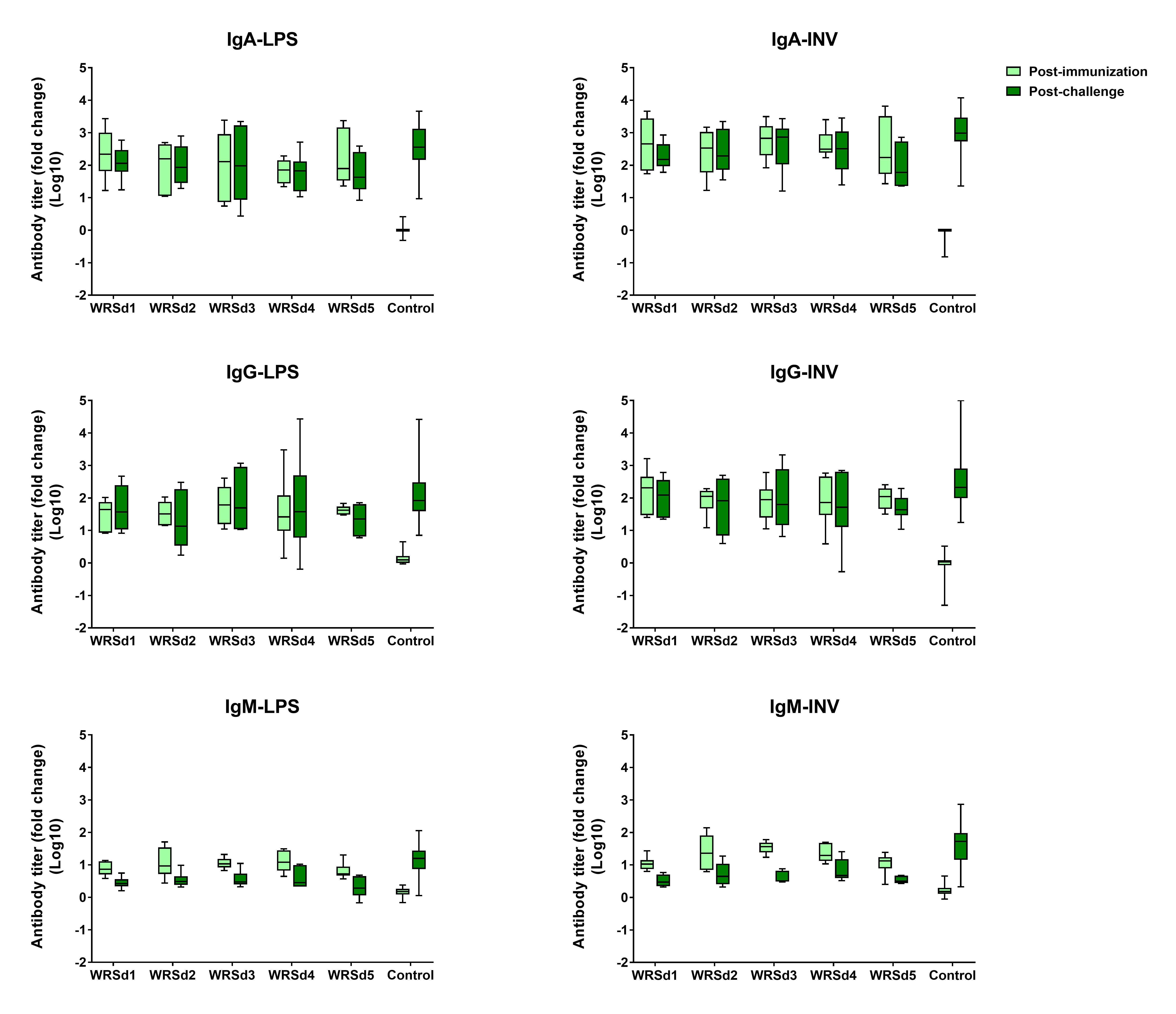

Supplement: Supplementary Figure S2 — The peak fold increase in serum antibody titers against the Sd1 LPS and INV antigens over baseline for the control and five vaccinated groups. Box and whisker plots represent the mean from 6 vaccinated monkeys and 18 control monkeys. Monkeys were immunized with the Sd1 vaccine strains on days 0, 3, and 6. On day 37, all animals were challenged with the Sd1 1617 wild-type strain. No significance was observed between post-immunization and post-challenge using the Mann-Whitney test. [file Image_2.TIF]

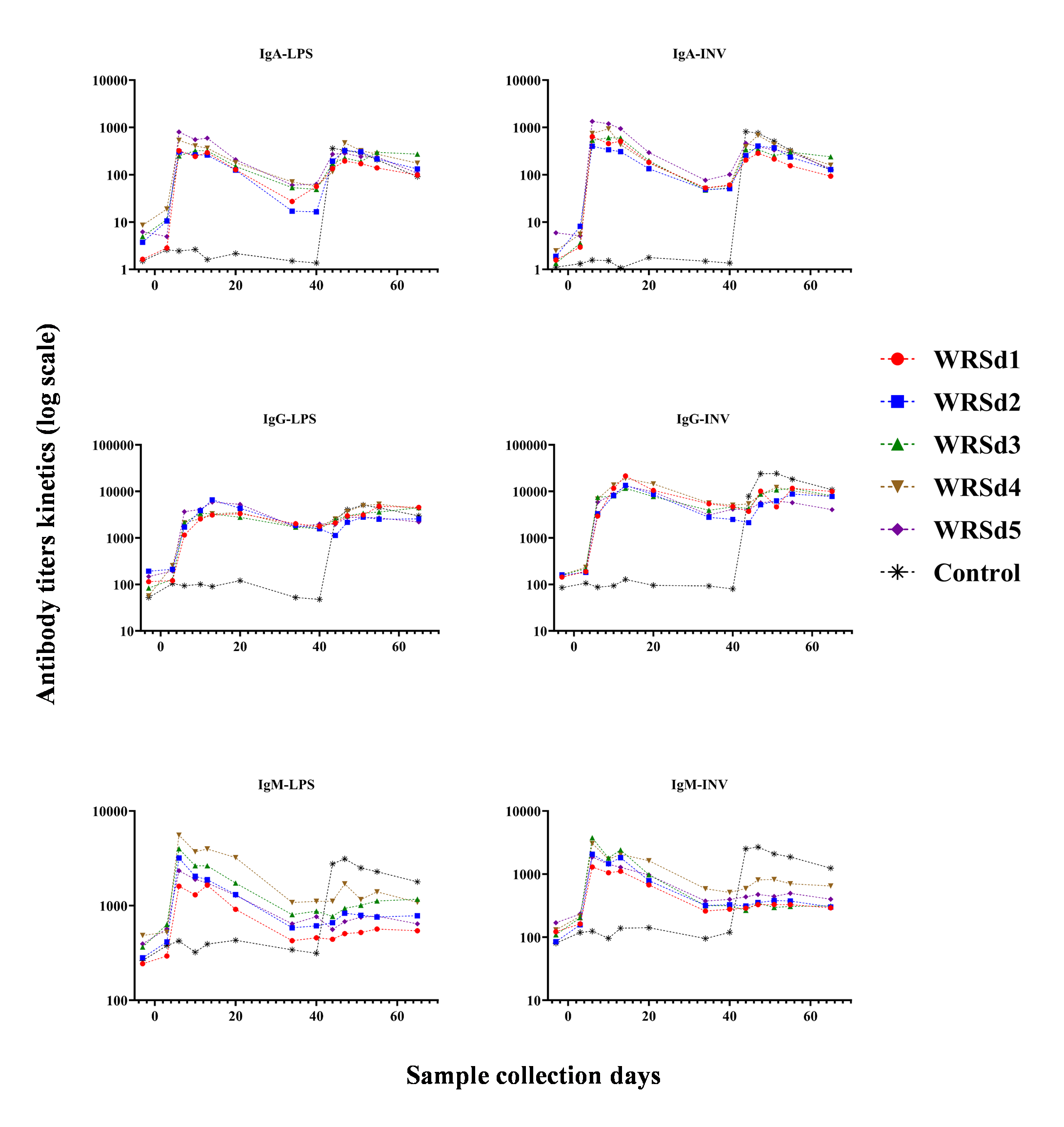

Supplement: Supplementary Figure S3 — The serum IgA, IgG, and IgM antibody titers kinetics against the Sd1 LPS and INV antigens were used for the control group and five vaccinated groups. Each symbol in each graph represents the geomean from 6 vaccinated monkeys and 18 control monkeys. Monkeys were immunized with the Sd1 vaccine strains on days 0, 3, and 6. On day 37, all animals were challenged with the Sd1 1617 wild-type strain. [file Image_3.TIF]

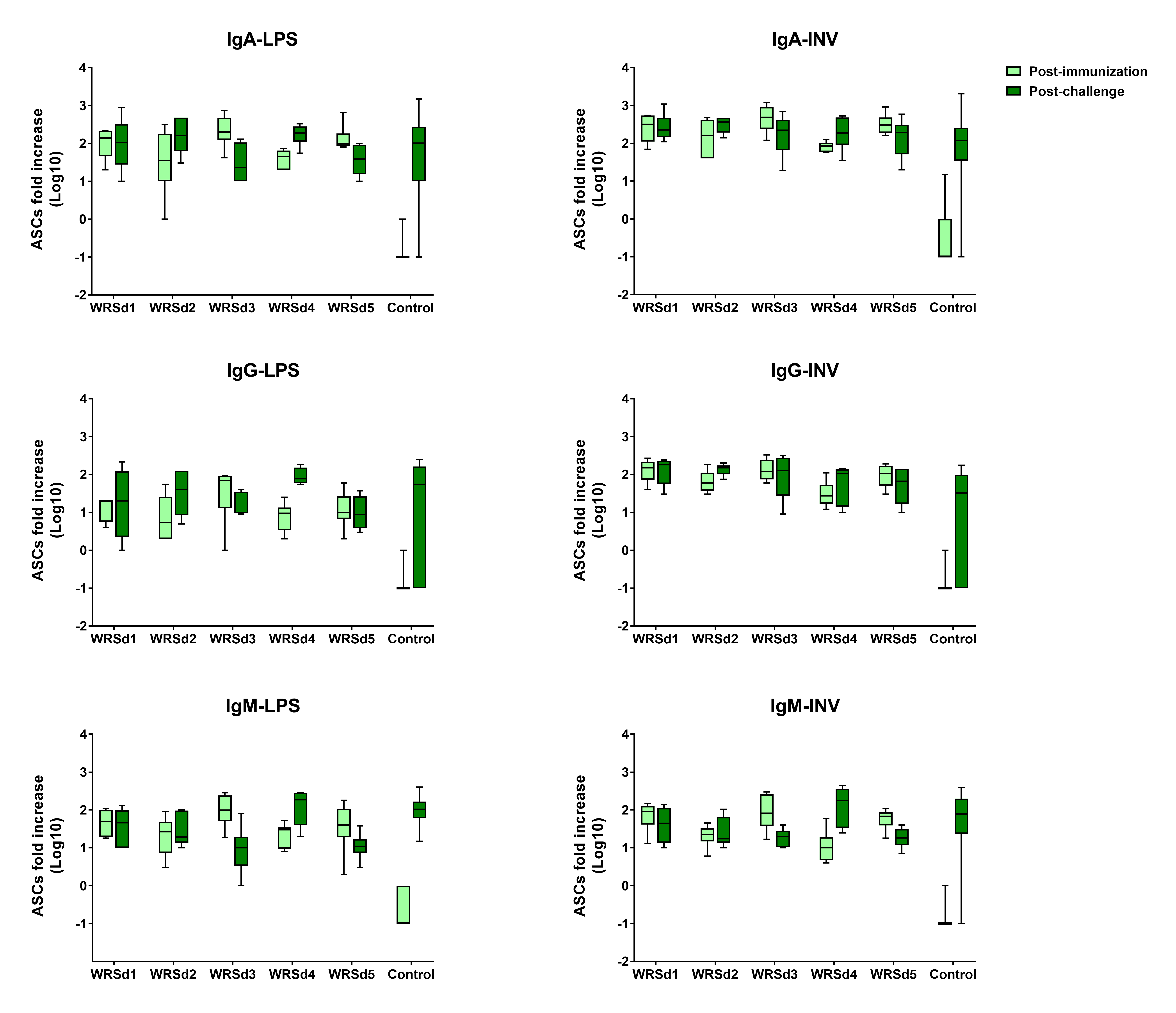

Supplement: Supplementary Figure S4 — The fold increase in ASC responses against the Sd1 LPS and INV antigens over baseline for the control and five vaccinated groups. Box and whisker plots represent the mean from 6 vaccinated monkeys and 18 control monkeys. Monkeys were immunized with the Sd1 vaccine strains on days 0, 3, and 6. On day 37, all animals were challenged with the Sd1 1617 wild-type strain. No significance was observed between post-immunization and post-challenge using the Mann–Whitney test. [file Image_4.TIF]

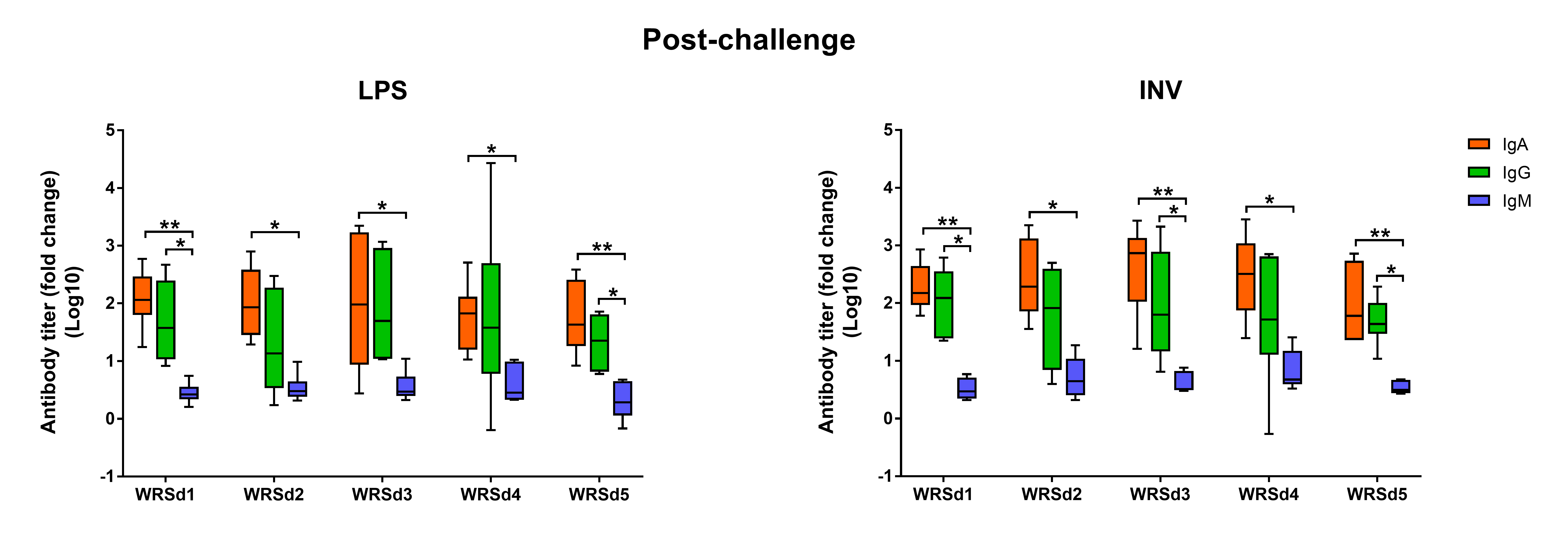

Supplement: Supplementary Figure S5 — Peak fold increase in IgA, IgG, and IgM antibody titers t post-challenge against Sd1 LPS and INV antigens for the five vaccinated groups. Monkeys were immunized with the Sd1 vaccine strains on days 0, 3, and 6. On day 37, all animals were challenged with the Sd1 1617 wild-type strain. The box and whisker plots represent the mean from six vaccinated monkeys. *p < 0.05, **p < 0.01, using the Kruskal–Wallis test followed using Dunn's post hoc test. [file Image_5.TIF]
